# Diabetic Peripheral Neuropathy Affects Pinch Strength and Hand Dexterity in Elderly Patients

**DOI:** 10.1155/2021/9959103

**Published:** 2021-07-20

**Authors:** Qi Zhang, Yifang Lin, Xinhua Liu, Li Zhang, Yan Zhang, Dong Zhao, Qi Lu, Jie Jia

**Affiliations:** ^1^Department of Rehabilitation Medicine, Huashan Hospital, Fudan University, China; ^2^Jiaozuo People's Hospital, Henan Province, China; ^3^National Clinical Research Center for Aging and Medicine, Huashan Hospital, Fudan University, China

## Abstract

**Objective:**

Diabetic peripheral neuropathy (DPN) is one of the most common chronic complications of diabetes, leading to disability and decreased quality of life. In past research and clinical studies, the lower limb function of DPN patients was often the principal subject of research, with little attention given to the upper limb and hand. Our goal was to assess and compare hand function between elderly diabetic patients with DPN and without DPN.

**Methods:**

A total of 52 diabetic patients were registered and underwent hand function assessments and electrodiagnostic tests. Dynamometer, pinch meter, Semmes Weinstein monofilaments, and the Purdue Pegboard Test (PPT) were used to assess the patients' grip strength, pinch strength, tactile sensory threshold, and hand dexterity.

**Results:**

Compared with the non-DPN group, the elderly DPN group showed worse thumb-middle fingertip pinch strength and thumb-little fingertip pinch strength in the dominant hand (3.50 (2.50, 4.25) vs. 4.50 (3.00, 5.00), *p* = 0.019; 1.50 (1.00, 2.00) vs. 2.50 (2.00, 3.00), *p* < 0.001); the elderly DPN group displayed worse thumb-middle fingertip pinch strength, thumb-ring fingertip pinch strength, and thumb-little fingertip pinch strength in the nondominant hand (3.50 (2.00, 4.50) vs. 4.00 (3.00, 5.00), *p* = 0.013; 2.50 (1.25, 3.00) vs. 3.00 (2.50, 3.50), *p* = 0.033; 1.00 (0.75, 2.25) vs. 2.50 (2.00, 2.50), *p* < 0.001). The elderly DPN group scored lower than the non-DPN group on the PPT test of assembly (13.96 ± 5.18 vs. 16.96 ± 4.61, *t* = 2.212, *p* = 0.032).

**Conclusion:**

Motor function limitation is the principal hand dysfunction in elderly patients with DPN, which is mainly manifested as a decline in fingertip pinch strength and a decrease in hand dexterity. This trial is registered with Clinical Trial Registry no. ChiCTR1900025358.

## 1. Introduction

China has the largest number of diabetes mellitus cases and the highest annual increases in cases in the world, with 116 million cases nationwide in 2019 [1, 2]. Diabetic peripheral neuropathy (DPN) is one of the most common chronic complications of diabetes, leading to disability and decreased quality of life, and its incidence increases with the duration of diabetes [3]. Approximately 10% to 15% of patients have signs of neuropathy when they are diagnosed with diabetes, and 50% of diabetics will eventually develop DPN, which could affect 236 million people worldwide by 2030 [4, 5]. DPN can affect both sensory neurons and motor neurons and is characterized by decreases in tactile sensitivity, nerve conduction velocity, and motor function in the advanced stages of the disease, which may eventually lead to foot ulcers, amputation, and disability [6, 7]. The major symptom of DPN is symmetrical sensory pain affecting the lower limbs [8]. Other manifestations include atypical pain, numbness, and pins-and-needles and hot or burning sensations. Other signs of neuromotor dysfunction include muscle weakness, poor balance, and a tendency to fall [9, 10].

With the increasing prevalence of diabetes and the prolongation of the life span of diabetic patients, the prevalence of diabetes in the elderly is rising [11]. Therefore, elderly people are also the main population affected by DPN. Previous researchers have focused on the lower limbs of patients with DPN but ignored the impact of DPN on the hands. A few studies have reported pain, numbness, and even ulcers in the hands of parents with DPN [12, 13]. Evidence has suggested that hand function, especially pinch and grip strength, decreases in type 2 diabetes mellitus compared with healthy controls [14]. However, limited studies have focused on sensory and motor function in the hands of elderly patients with DPN. Based on previous studies, we assume that elderly DPN patients have decreased sensory and motor function compared to non-DPN patients. The aim of this study was to document the monofilament tactile sensation, grip strength, pinch strength, and dexterity of the hands of the elderly patients with DPN and to explore the rehabilitation strategies for hand function in DPN patients.

## 2. Research Design and Methods

### 2.1. Study Design and Participants

**Figure 1:**
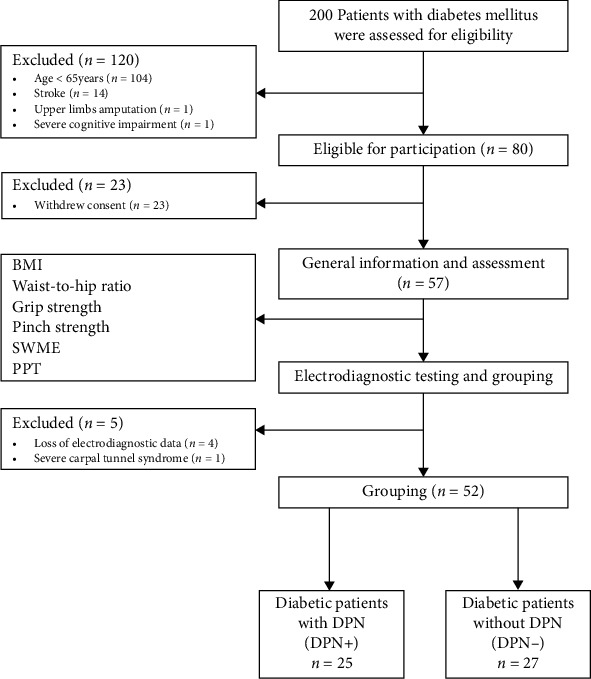
Participant inclusion criteria and research procedure. SWME: Semmes Weinstein monofilament examination; PPT: Purdue Pegboard Test.

**Figure 2:**
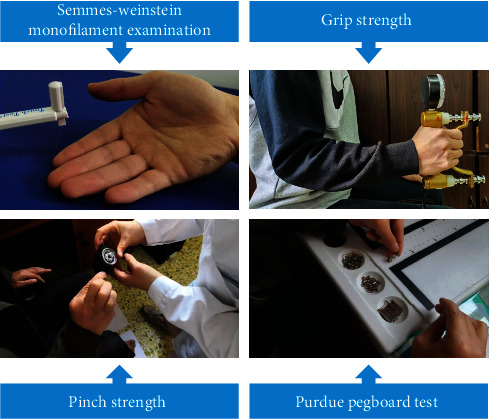
Illustration of hand function assessment.

Data were collected in Jiaozuo People's Hospital, Henan Province, China. The inclusion criteria were as follows: (1) participants were age 65 years or older with a previous diagnosis of type 2 diabetes for more than 5 years; (2) their blood glucose control was basically stable, and there was no acute complications of diabetes; and (3) informed consent was obtained from all participants. The exclusion criteria included the following: (1) history of stroke, Parkinson's disease, and other central system diseases; (2) history of peripheral nervous system diseases such as carpal tunnel syndrome; (3) hands with ulcers, gout, and disability; (4) new fractures and severe muscle atrophy in the hands; (5) habit/history of alcohol abuse; (6) participants with no education; and (7) participants with severe cognitive impairment (Montreal Cognitive Assessment (MoCA) score ≤ 18). This study was approved by the Ethics Committee of Huashan Hospital Affiliated to Fudan University (NO.2019M-006) and the Ethics Committee of Jiaozuo People's Hospital (NO.2019001) and was registered in the China Clinical Trial Registration Center (ChiCTR1900025358). A total of 52 participants who met the above criteria were included (Figure 1). All participants were included in strict accordance with our standards, and general information, hand function assessment, and electrophysiological diagnosis were gathered from all participants who met the standards in accordance with specific procedures (Figure 2). All participants were divided into two groups according to whether they had DPN: elderly diabetic patients with DPN (DPN+) and elderly diabetic patients without DPN (DPN-).

### 2.2. General Information

Data on all participants included age, sex, height, weight, waist circumference, hip circumference, diabetes duration, and medical history.

### 2.3. Monofilament Tactile Sensation Assessment

**Figure 3:**
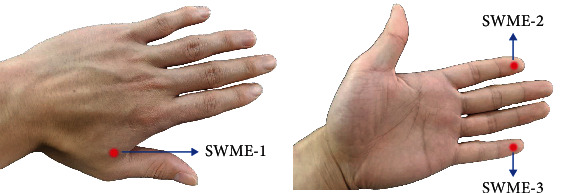
The assessment sites for the Semmes Weinstein monofilament examination.

Semmes Weinstein monofilament examination (SWME) is often used to assess the tactile sensation function of the hands and feet in patients with DPN [15]. All participants were asked to wear eye masks for the Semmes Weinstein monofilament assessment (Touch Test Complete Hand Kit, North Coast Medical Inc., USA) following a standard testing protocol [16]. We assessed the area innervated by the median, ulnar, and radial nerves: the dorsal area of the first and second metacarpal bones (SWME-1), the tip of the index finger (SWME-2), and the tip of the little finger (SWME-3). Each part was assessed three times, and the minimum tactile threshold that the participant could feel was recorded (Figure 3).

### 2.4. Grip and Pinch Strength Assessment of Hands

Grip strength of the participants was assessed using a hydraulic hand dynamometer (Exacta, North Coast Medical Inc., Gilroy, CA, USA) with participants seated with the shoulder adducted and neutrally rotated, the elbow flexed at 90 degrees, and the forearm in the neutral position. We used a mechanical pinch-strength meter (B&L Engineering, CA, United States) to measure the pinch strength between the thumb and the remaining four fingers. The measurement methods were as follows: (1) thumb-index fingertip pinch strength (Pinch-I), (2) thumb-middle fingertip pinch strength (Pinch-M), (3) thumb-ring fingertip pinch strength (Pinch-R), and (4) thumb-little fingertip pinch strength (Pinch-L). Each was measured three times, and the maximum value was recorded.

### 2.5. Assessment of Hand Dexterity

Hand dexterity is an important manifestation of upper limb motor function. We also assessed fine-motor-skill performance using the previously validated Purdue Pegboard Test (PPT, model 32020, Lafayette Instruments, Lafayette, IN, USA) following a standard testing protocol [17]. Participants were asked to complete four tests: (1) fill in the holes with pegs within 30 seconds with the dominant hand, (2) fill in the holes with pegs within 30 seconds with the nondominant hand, (3) fill in the holes with pegs within 30 seconds with the both hands, and (4) assemble in sequence a peg, a washer, a collar, and finally another washer within 60 seconds. The number of parts completed by participants was recorded.

### 2.6. Nerve Conduction Studies

Nerve conduction studies (NCS) are the current gold standard for the diagnosis of DPN [18]. All participants underwent NCS using a standard electromyography (EMG) device (KOHDEN, MEB-9200K, Japan). Testing was performed by a professional technician with more than 10 years of clinical experience in the EMG room, and the entire process was conducted in a special shielded room. There were 32 variables recorded: sensory nerve conduction velocity (SNCV) and sensory nerve action potential (SNAP) in the median, ulnar, sural, and superficial peroneal nerves on both sides; motor nerve conduction velocity (MNCV) and compound muscle action potential (CMAP) in the median, ulnar, peroneal, and tibial nerves on both sides. We used NCS to diagnose DPN using the criteria for electrodiagnostic confirmation of DPN: an abnormality of any attribute of nerve conduction in two separate nerves, one of which must be a nerve in the lower limbs [19, 20].

### 2.7. Statistical Analysis

Statistical analysis was performed using SPSS for Windows, version 21.0 (SPSS, Chicago, IL). All statistics were tested for normal distribution. The continuous data of normal distribution were expressed as mean ± standard deviation, and categorical variables were presented as numbers and percentages (%). An independent sample *t*-test was used to compare the means of two groups, and a chi-square test was used to compare the percentages. Data of skewed distribution were presented as the median (25th-75th percentile) and tested with the Mann–Whitney *U* test. A value of *p* < 0.05 was considered statistically significant.

## 3. Results

### 3.1. Baseline Characteristics of DPN and Non-DPN Groups

A total of 52 participants were included in our study. The DPN group had 25 participants (12 males, 13 females) with an average age of 73.0 ± 5.3 years. The body mass index (BMI) was 25.6 ± 3.0 kg/m^2^, the waist-to-hip ratio was 0.93 ± 0.07, and the diabetes duration was 15.0 (9.5, 21.0) years. The percentage of participants who self-reported smoking, drinking, and coronary heart disease was 28.0%, 24.0%, and 40.0%, respectively. The non-DPN group had 27 participants (8 males, 19 females) with an average age of 72.1 ± 5.5 years. The BMI was 25.5 ± 3.6 kg/m^2^, the waist-to-hip ratio was 0.90 ± 0.07, and the diabetes duration was 12.0 (6.0, 16.0) years. The percentage of participants who self-reported smoking, drinking, and coronary heart disease was 11.1%, 11.1%, and 37.0%, respectively. There were no significant differences in baseline data between the two groups (*p* > 0.05) (Table 1).

### 3.2. NCS and Monofilament Tactile Threshold Analysis

In the NCS results, there were significant differences in the nerve conduction velocities in both hands between the DPN group and the non-DPN group. The results showed that the conduction velocities in the median sensory nerve, median motor nerve, and ulnar sensory nerve in the dominant hand in the DPN group were lower than those in the non-DPN group (*p* < 0.05). The conduction velocities in the median sensory nerve and median motor nerve in the nondominant hand in the DPN group were lower than those in the non-DPN group (*p* < 0.05). There were significant differences in four nerve conduction velocities in the lower limbs between the two groups (*p* < 0.05). The results showed that the median sensory nerve action potential, the peroneal nerve compound muscle action potential, and the tibial nerve compound muscle action potential in the dominant hand/lower limb in the DPN group were lower than those in the non-DPN group (*p* < 0.05). The median sensory nerve action potential, the median nerve compound muscle action potential, the ulnar sensory nerve action potential, and the peroneal nerve compound muscle action potential in the nondominant hand/lower limb in the DPN group were lower than those of the non-DPN group (*p* < 0.05). There were no significant differences in the SWME threshold between the two groups (*p* > 0.05) (Table 2).

### 3.3. Analysis of Hand Grip and Pinch Strength

**Figure 4:**
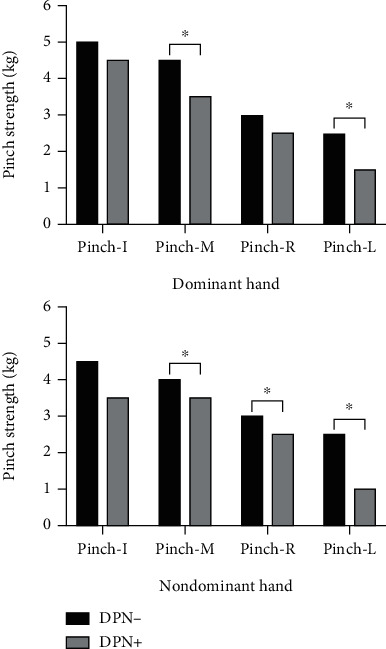
Differences in pinch strength between the thumb and the other four fingers in both hands. Pinch-I: thumb-index fingertip pinch strength; Pinch-M: thumb-middle fingertip pinch strength; Pinch-R: thumb-ring fingertip pinch strength; Pinch-L: thumb-little fingertip pinch strength. ^∗^A *p* value < 0.05 was considered to indicate statistical significance.

Analysis of hand grip strength of all participants showed no significant differences between the two groups (*p* > 0.05). The results showed that the thumb-middle fingertip pinch strength and thumb-little fingertip pinch strength of the DPN group were lower than those of the non-DPN group in the dominant hand (*p* < 0.05). There were no significant differences in the thumb-index fingertip pinch strength and thumb-ring fingertip pinch strength between the two groups in the dominant hand (*p* > 0.05). The thumb-middle fingertip pinch strength, thumb-ring fingertip pinch strength, and thumb-little fingertip pinch strength of the DPN group were lower than those of the non-DPN group in the nondominant hand (*p* < 0.05) (Table 3; Figure 4).

### 3.4. Analysis of Hand Dexterity

**Figure 5:**
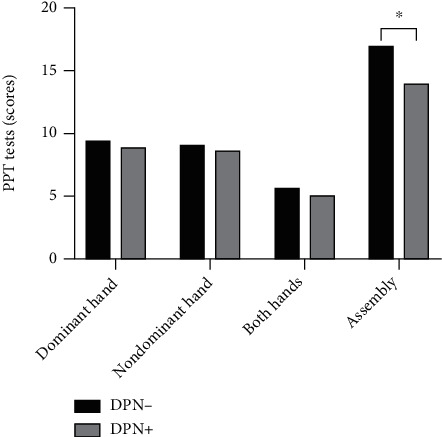
Differences between the two Purdue Pegboard Tests (PPT). ^∗^A *p* value < 0.05 was considered to indicate statistical significance.

Table 4 shows PPT differences in the dominant hand, nondominant hand, both hands, and assembly between the DPN group and the non-DPN group. There were no significant differences between the two groups of participants in the PPT test of dominant hand, nondominant hand, and both hands (*p* > 0.05). The results showed that the DPN group scored lower than the non-DPN group in PPT test of assembly (13.96 ± 5.18 vs. 16.96 ± 4.61, *t* = 2.212, *p* = 0.032) (Table 4; Figure 5).

## 4. Discussion

The study showed that elderly patients with DPN displayed worse fingertip pinch strength, especially in thumb-middle fingertip pinch strength, thumb-ring fingertip pinch strength, and thumb-little fingertip pinch strength in both dominant and nondominant hands. Weakness in pinch strength may affect the dexterity of the patient's hands.

Sensory function is an important part of hand function. In the past reports, DPN caused sensory impairments such as pain, decreased vibration sensation, and loss of tactile sensation [21, 22]. Sensory function is closely related to peripheral nerves. SWME is often used to assess the tactile sensation in patients with DPN [23]. In our study, there were no significant differences between the SWME assessment results of the DPN group and the non-DPN group. A possible explanation for this might be that the degree of upper and lower limb nerve damage was not consistent. Liu et al. performed EMG assessment on 700 DPN patients and found that the nerve conduction abnormality in the lower limbs was more serious than that in the upper limbs [24]. In our study, we found that peripheral nerves in the lower limbs seem to be more likely to be affected (Table 2). This suggests that peripheral neuropathy in the upper limbs of DPN patients was not serious compared with that in the lower limbs. Moreover, the tactile sensation assessed by SWME pertains to large-fiber function [23]. In the early stage of DPN development, the small fibers of the peripheral nerves are damaged first, followed by gradual damage in the large fibers of the nerve [25, 26]. This also may explain why SWME could not accurately assess the hand tactile sensation in DPN patients.

Grip strength is a measure of upper limb performance and is closely related to muscle strength, which is considered an important component of physical fitness and a convenient but powerful predictor of disability, morbidity, and mortality in the elderly population [27–29]. The results of this study showed no significant differences in hand grip strength between the DPN group and the non-DPN group. This is consistent with the results of Lima et al., which showed that mild to moderate DPN had no significant effect on maximum hand grip strength [30]. The reason for this result might be due to factors such as the older age and the longer diabetes duration in the two groups. In addition, sarcopenia, which is caused by risk factors such as diabetes, high blood pressure, and dyslipidemia, leads to a decrease in skeletal muscle mass, muscle strength, and physical function in older adults [31]. Compared with DPN, sarcopenia might have a greater impact on grip strength.

Pinch strength is the most frequently used hand motor function in activities of daily living, and it is also an important part of hand function. Good pinch strength is conducive to the fine motor movements of the hands. In this study, a fingertip-type pinch strength measurement method was used, and it was found that the hand pinch strength performance of the DPN group was worse than that of the non-DPN group, especially the thumb-middle fingertip pinch strength, thumb-ring fingertip pinch strength, and thumb-little fingertip pinch strength in both hands (Figure 4). This difference might be due to skeletal muscle atrophy caused by distal peripheral neuropathy, which may lead to the decrease in hand pinch strength in DPN patients. In a prior study, Anderson et al. performed MRI on the lower leg muscles of DPN patients and found that distal muscle atrophy was the most obvious [32]. This kind of skeletal muscle atrophy caused by peripheral neuropathy may be present not only in the lower limbs but also in the upper limbs and hands.

Reduced hand dexterity will directly affect the hand function of the elderly and indirectly lead to the decline in activities of daily living. In this study, we tested hand dexterity using PPT and found no differences between the two groups when DPN patients performed relatively simple hole-filling tasks. When patients performed assembly activities, the DPN group was worse than the non-DPN group (Figure 5). This result might be explained by the fact that the pinch strength of the patient's hand was decreased, which affected the fine movement and dexterity of the DPN patients' hands. In addition, there is evidence that the cognitive function of DPN patients is worse, mainly in executive function, which may affect the patient's left-right coordination [33]. Because the PPT test requires the patient to have a certain level of cognitive function (executive function) to complete the assembly, the assembly task performance of the DPN group was worse.

In our clinical work, although the lower limb dysfunction of DPN patients was prominent, function problems in the upper limbs and hands could not be ignored. In the future rehabilitation training, the sensory and motor functions of the hands of patients with DPN deserve clinical attention by therapists. In particular, focusing on the patient's hand muscle strength and dexterity may improve the patient's quality of life. There are some limitations in this study. The sample is small and a cross-sectional study with a single source of cases. Therefore, the results obtained may be biased and relatively limited. In the future, large-sample, multicenter research will need to be carried out, and a control group will be set up. In addition, sensory assessment was relatively narrow. We focused only on the tactile sensation of DPN patients and did not assess patients' temperature sensation, vibration sensation, and two-point discrimination.

## 5. Conclusions

In the elderly, limited motor function is the main hand dysfunction in patients with DPN, which is mostly manifested as a decline of fingertip pinch strength and decrease in hand dexterity.

## Figures and Tables

**Table 1:** Characteristics of the DPN group (DPN+) and the non-DPN group (DPN-).

	DPN group (DPN+)	Non-DPN group (DPN-)	*t*/*X*^2^/*Z*	*p*
*N*	25	27		
Age (years)	73.0 ± 5.3	72.1 ± 5.5	-0.568	0.573
Male/female (number)	12/13	8/19	1.851	0.174
BMI (kg/m^2^)	25.6 ± 3.0	25.5 ± 3.6	-0.123	0.903
Waist-to-hip ratio	0.93 ± 0.07	0.90 ± 0.07	-1.587	0.119
Diabetes duration (years)	15.0 (9.5, 21.0)	12.0 (6.0, 16.0)	-1.714	0.086
Current smoker, *n* (%)	7 (28.0%)	3 (11.1%)	2.384	0.123
Current drinker, *n* (%)	6 (24.0%)	3 (11.1%)	1.507	0.220
Coronary heart disease, *n* (%)	10 (40.0%)	10 (37.0%)	0.048	0.826

Data are expressed as the mean ± standard deviation, %, or median (25th-75th percentile). BMI: body mass index. A *p* value > 0.05 was considered as not indicating statistical significance.

**Table 2:** Comparison of nerve conduction studies and Semmes Weinstein monofilament examination threshold between the two groups.

	Dominant hand/lower limb				Nondominant hand/lower limb			
	DPN+	DPN-	*t*/*Z*	*p*	DPN+	DPN-	*t*/*Z*	*p*
M-SNCV (m/s)	46.4 ± 6.4	49.8 ± 4.8	2.100	0.041^∗^	45.5 ± 6.8	52.0 ± 4.9	3.836	<0.001^∗^
M-MNCV (m/s)	49.1 ± 4.7	52.2 ± 4.2	2.458	0.018^∗^	50.1 ± 5.8	53.7 ± 4.9	2.357	0.023^∗^
U-SNCV (m/s)	47.2 ± 4.5	49.9 ± 3.0	2.537	0.014^∗^	47.7 ± 5.2	50.2 ± 4.3	1.849	0.071
U-MNCV (m/s)	53.6 ± 7.3	56.2 ± 4.9	1.509	0.138	55.1 ± 6.9	56.1 ± 5.1	0.619	0.539
S-SNCV (m/s)	42.2 ± 4.8	46.7 ± 4.1	3.451	0.001^∗^	42.0 ± 6.1	46.6 ± 5.0	2.840	0.007^∗^
SP-SNCV (m/s)	41.5 ± 6.1	46.4 ± 4.3	3.189	0.003^∗^	41.1 ± 6.3	45.3 ± 3.7	2.869	0.006^∗^
P-MNCV (m/s)	41.0 ± 4.8	44.6 ± 4.1	2.880	0.006^∗^	40.2 ± 5.4	43.7 ± 2.9	2.858	0.006^∗^
T-MNCV (m/s)	40.2 ± 3.7	43.1 ± 3.1	3.025	0.004^∗^	39.6 ± 4.6	43.5 ± 2.9	3.655	0.001^∗^
M-SNAP (*μ*V)	4.9 ± 3.9	9.5 ± 7.7	2.762	0.009^∗^	6.7 ± 5.4	12.2 ± 5.3	3.681	0.001^∗^
M-CMAP (mV)	8.1 ± 4.4	9.0 ± 2.9	0.926	0.359	8.4 ± 4.3	10.7 ± 3.3	2.136	0.038^∗^
U-SNAP (*μ*V)	7.4 ± 5.9	9.4 ± 6.8	1.125	0.266	5.8 ± 4.9	9.8 ± 6.2	2.530	0.015^∗^
U-CMAP (mV)	11.5 ± 4.1	12.1 ± 2.9	0.578	0.566	10.7 ± 4.2	12.2 ± 2.2	1.704	0.095
S-SNAP (*μ*V)	8.8 ± 9.8	12.1 ± 9.5	1.222	0.228	8.9 ± 12.3	8.6 ± 4.7	-0.136	0.893
SP-SNAP (*μ*V)	6.1 ± 9.3	11.0 ± 10.4	1.784	0.080	6.9 ± 10.6	11.4 ± 8.5	1.645	0.107
P-CMAP (mV)	3.8 ± 2.2	6.5 ± 2.6	4.106	<0.001^∗^	3.8 ± 2.7	6.9 ± 3.6	3.547	0.001^∗^
T-CMAP (mV)	10.7 ± 6.7	14.9 ± 7.2	2.161	0.036^∗^	9.9 ± 5.2	12.9 ± 6.4	1.858	0.069
SWME-1	2.83 (2.44, 3.61)	2.83 (2.36, 3.61)	-0.751	0.453	2.83 (2.36, 3.22)	2.83 (2.36, 3.22)	-0.308	0.758
SWME-2	3.61 (2.83, 3.73)	2.83 (2.44, 3.61)	-1.074	0.283	2.83 (2.44, 3.61)	2.83 (2.44, 3.22)	-0.981	0.327
SWME-3	3.22 (2.83, 3.73)	2.83 (2.44, 3.22)	-1.453	0.146	2.83 (2.44, 3.61)	2.83 (2.44, 3.22)	-1.377	0.168

Data are expressed as the mean ± standard deviation or median (25th-75th percentile). M-SNCV: median sensory nerve conduction velocity; M-MNCV: median motor nerve conduction velocity; U-SNCV: ulnar sensory nerve conduction velocity; U-MNCV: ulnar motor nerve conduction velocity; S-SNCV: sural sensory nerve conduction velocity; SP-SNCV: superficial peroneal sensory nerve conduction velocity; P-MNCV: peroneal motor nerve conduction velocity; T-MNCV: tibial motor nerve conduction velocity; M-SNAP: median sensory nerve action potential; M-CMAP: median nerve compound muscle action potential; U-SNAP: ulnar sensory nerve action potential; U-CMAP: ulnar nerve compound muscle action potential; S-SNAP: sural sensory nerve action potential; SP-SNAP: superficial peroneal sensory nerve action potential; P-CMAP: peroneal nerve compound muscle action potential; T-CMAP: tibial nerve compound muscle action potential. ^∗^A *p* value < 0.05 was considered to indicate statistical significance.

**Table 3:** Comparison of grip and pinch strength between the two groups.

	Dominant hand				Nondominant hand			
	DPN+	DPN-	*Z*	*p*	DPN+	DPN-	*Z*	*p*
Grip strength (kg)	16.00 (12.50, 26.50)	17.00 (14.00, 24.00)	-0.340	0.734	15.00 (8.50, 23.00)	16.00 (12.00, 21.00)	-0.468	0.640
Pinch-I (kg)	4.50 (3.50, 5.50)	5.00 (4.00, 6.50)	-1.482	0.138	3.50 (2.75, 5.25)	4.50 (3.50, 6.00)	-1.821	0.069
Pinch-M (kg)	3.50 (2.50, 4.25)	4.50 (3.00, 5.00)	-2.341	0.019^∗^	3.50 (2.00, 4.50)	4.00 (3.00, 5.00)	-2.489	0.013^∗^
Pinch-R (kg)	2.50 (1.50, 3.50)	3.00 (2.50, 4.00)	-1.878	0.060	2.50 (1.25, 3.00)	3.00 (2.50, 3.50)	-2.134	0.033^∗^
Pinch-L (kg)	1.50 (1.00, 2.00)	2.50 (2.00, 3.00)	-4.046	<0.001^∗^	1.00 (0.75, 2.25)	2.50 (2.00, 2.50)	-3.689	<0.001^∗^

Data are expressed as the median (25th-75th percentile). Pinch-I: thumb-index fingertip pinch strength; Pinch-M: thumb-middle fingertip pinch strength; Pinch-R: thumb-ring fingertip pinch strength; Pinch-L: thumb-little fingertip pinch strength. ^∗^A *p* value < 0.05 was considered to indicate statistical significance.

**Table 4:** Comparison of the scores on the Purdue Pegboard Test (PPT) between the two groups.

	DPN group (DPN+)	Non-DPN group (DPN-)	*t*	*p*
PPT (dominant hand)	8.88 ± 1.90	9.37 ± 2.05	0.890	0.378
PPT (nondominant hand)	8.56 ± 2.22	9.04 ± 1.56	0.903	0.371
PPT (both hands)	5.04 ± 1.59	5.63 ± 1.52	1.364	0.179
PPT (assembly)	13.96 ± 5.18	16.96 ± 4.61	2.212	0.032^∗^

Data were expressed the mean ± standard deviation. ^∗^A *p* value < 0.05 was considered to indicate statistical significance.

## Data Availability

Datasets analyzed during the current study are available from the corresponding author on reasonable request.

## References

[1] Saeedi P., Petersohn I., Salpea P. (2019). Global and regional diabetes prevalence estimates for 2019 and projections for 2030 and 2045: results from the International Diabetes Federation Diabetes Atlas, 9^th^ edition. *Diabetes Research and Clinical Practice*.

[2] Ma R. C. W. (2018). Epidemiology of diabetes and diabetic complications in China. *Diabetologia*.

[3] Iqbal Z., Azmi S., Yadav R. (2018). Diabetic peripheral neuropathy: epidemiology, diagnosis, and pharmacotherapy. *Clinical Therapeutics*.

[4] Määttä L. L., Charles M., Witte D. R. (2019). Prospective study of neuropathic symptoms preceding clinically diagnosed diabetic polyneuropathy: addition-Denmark. *Diabetes Care*.

[5] Tesfaye S., Selvarajah D. (2012). Advances in the epidemiology, pathogenesis and management of diabetic peripheral neuropathy. *Diabetes/Metabolism Research and Reviews*.

[6] Watkins P. J., Thomas P. K. (1998). Diabetes mellitus and the nervous system. *Journal of Neurology, Neurosurgery & Psychiatry*.

[7] Ramji N., Toth C., Kennedy J., Zochodne D. W. (2007). Does diabetes mellitus target motor neurons?. *Neurobiology of Disease*.

[8] Dworkin R. H., Connor A. B. O., Backonja M. (2007). Pharmacologic management of neuropathic pain: evidence-based recommendations. *Pain*.

[9] Backonja M.-M., Stacey B. (2004). Neuropathic pain symptoms relative to overall pain rating. *The Journal of Pain*.

[10] Khdour M. R. (2020). Treatment of diabetic peripheral neuropathy: a review. *Journal Of Pharmacy And Pharmacology*.

[11] Kalra S., Sharma S. K. (2018). Diabetes in the elderly. *Diabetes Therapy*.

[12] Wang C., Lv L., Wen X. (2010). A clinical analysis of diabetic patients with hand ulcer in a diabetic foot centre. *Diabetic Medicine*.

[13] Win M. M. T. M., Fukai K., Nyunt H. H., Hyodo Y., Linn K. Z. (2019). Prevalence of peripheral neuropathy and its impact on activities of daily living in people with type 2 diabetes mellitus. *Nursing & Health Sciences*.

[14] Gundmi S., Maiya A. G., Bhat A. K., Ravishankar N., Hande M. H., Rajagopal K. V. (2018). Hand dysfunction in type 2 diabetes mellitus: systematic review with meta-analysis. *Annals of physical and rehabilitation medicine*.

[15] Ennis S. L., Galea M. P., Neal D. N. O., Dodson M. J. (2016). Peripheral neuropathy in the hands of people with diabetes mellitus. *Diabetes Research and Clinical Practice*.

[16] Bell K., Tomancik J., Tomancik E. (1987). The Repeatability of Testing with Semmes-Weinstein Monofilaments. *The Journal of Hand Surgery*.

[17] Yang C.-J., Hsu H.-Y., Lu C.-H., Chao Y.-L., Chiu H.-Y., Kuo L.-C. (2015). The associations among hand dexterity, functional performance, and quality of life in diabetic patients with neuropathic hand from objective-and patient-perceived measurements. *Quality of Life Research*.

[18] Selvarajah D., Kar D., Khunti K. (2019). Diabetic peripheral neuropathy: advances in diagnosis and strategies for screening and early intervention. *The Lancet Diabetes & Endocrinology*.

[19] England J. D., Gronseth G. S., Franklin G. (2005). Distal symmetrical polyneuropathy: a definition for clinical research. A report of the American Academy of Neurology, the American Association of Electrodiagnostic Medicine, and the American Academy of Physical Medicine and Rehabilitation. *Archives Of Physical Medicine And Rehabilitation*.

[20] Höliner I. (2013). Validity of the neurological examination in diagnosing diabetic peripheral neuropathy. *Pediatric Neurology*.

[21] Jensen T. S. (2006). New perspectives on the management of diabetic peripheral neuropathic pain. *Diabetes and Vascular Disease Research*.

[22] Santos T. R. M., Melo J. V., Leite N. C., Salles G. F., Cardoso C. R. L. (2018). Usefulness of the vibration perception thresholds measurement as a diagnostic method for diabetic peripheral neuropathy: results from the Rio De Janeiro type 2 diabetes cohort study. *Journal of Diabetes and its Complications*.

[23] Kamei N., Yamane K., Nakanishi S. (2005). Effectiveness of Semmes-Weinstein monofilament examination for diabetic peripheral neuropathy screening. *Journal of Diabetes and its Complications*.

[24] Liu M. S., Hu B. L., Cui L. Y., Tang X. F., Du H., Li B. H. (2005). Clinical and neurophysiological features of 700 patients with diabetic peripheral neuropathy. *Zhonghua Nei Ke Za Zhi*.

[25] Pop-Busui R. (2017). Diabetic neuropathy: a position statement by the American Diabetes Association. *Diabetes Care*.

[26] Petropoulos I. N., Ponirakis G., Khan A., Almuhannadi H., Gad H., Malik R. A. (2018). Diagnosing diabetic neuropathy: something old, something new. *Diabetes & Metabolism Journal*.

[27] Visser M., Newman A. B., Nevitt M. C. (2000). Reexamining the sarcopenia hypothesis: muscle mass versus muscle strength. *Annals of the New York Academy of Sciences*.

[28] Mayhew A. J., Griffith L. E., Gilsing A., Beauchamp M. K., Kuspinar A., Raina P. (2020). The association between self-reported and performance-based physical function with activities of daily living disability in the Canadian Longitudinal Study on Aging. *The Journals of Gerontology: Series A*.

[29] Vancampfort D., Stubbs B., Firth J., Koyanagi A. (2019). Handgrip strength, chronic physical conditions and physical multimorbidity in middle-aged and older adults in six low- and middle income countries. *European Journal of Internal Medicine*.

[30] Lima A., Carvalho K., Borges L. D. S., Hatanaka E., Rolim L. C., de Freitas P. B. (2017). Grip force control and hand dexterity are impaired in individuals with diabetic peripheral neuropathy. *Neuroscience Letters*.

[31] Chen L.-K., Woo J., Assantachai P. (2020). Asian Working Group for Sarcopenia: 2019 Consensus update on sarcopenia diagnosis and treatment. *Journal of the American Medical Directors Association*.

[32] Andersen H., Gadeberg P. C., Brock B., Jakobsen J. (1997). Muscular atrophy in diabetic neuropathy: a stereological magnetic resonance imaging study. *Diabetologia*.

[33] Rucker J. L., Jernigan S. D., McDowd J. M., Kluding P. M. (2014). Adults with diabetic peripheral neuropathy exhibit impairments in multi-tasking and other executive functions. *Journal of neurologic physical therapy: JNPT*.

